# Use of Antimetastatic SOD3-Mimetic Albumin as a Primer in Triple Negative Breast Cancer

**DOI:** 10.1155/2019/3253696

**Published:** 2019-02-28

**Authors:** Shanta M. Messerli, Amanda M. Schaefer, Yongxian Zhuang, Bohdan J. Soltys, Noah Keime, Jenny Jin, Li Ma, Carleton J. C. Hsia, W. Keith Miskimins

**Affiliations:** ^1^Cancer Biology and Immunotherapies, Sanford Research, Sioux Falls, 57104 SD, USA; ^2^AntiRadical Therapeutics LLC, Sioux Falls, 57104 SD, USA; ^3^Department of Physics, Georgia Southern University, Statesboro, GA 30460, USA

## Abstract

Of the deaths attributed to cancer, 90% are due to metastasis. Treatments that prevent or cure metastasis remain elusive. Low expression of extracellular superoxide dismutase (EcSOD or SOD3) has been associated with poor outcomes and increased metastatic potential in multiple types of cancer. Here, we characterize the antimetastatic therapeutic mechanisms of a macromolecular extracellular SOD3-mimetic polynitroxyl albumin (PNA, also known as VACNO). PNA is macromolecular human serum albumin conjugated with multiple nitroxide groups and acts as an SOD-mimetic. Here we show that PNA works as a SOD3-mimetic in a highly metastatic 4T1 mouse model of triple negative breast cancer (TNBC).* In vitro*, PNA dose dependently inhibited 4T1 proliferation, colony formation, and reactive oxygen species (ROS) formation.* In vivo*, PNA enhanced reperfusion time in the hypoxic cores of 4T1 tumors as measured by ultrasound imaging. Furthermore, PNA enhanced ultrasound signal intensity within the cores of the 4T1 tumors, indicating PNA can increase blood flow and blood volume within the hypoxic cores of tumors. Lung metastasis from 4T1 flank tumor was inhibited by PNA in the presence or absence of doxorubicin, a chemotherapy agent that produces superoxide and promotes metastasis. In a separate study, PNA increased the survival of mice with 4T1 flank tumors when used in conjunction with three standard chemotherapy drugs (paclitaxel, doxorubicin, and cyclophosphamide), as compared to treatment with chemotherapy alone. In this study, PNA-increased survival was also correlated with reduction of lung metastasis. These results support the hypothesis that PNA works through the inhibition of extracellular superoxide/ROS production leading to the conversion of 4T1 cells from a metastatic tumorigenic state to a cytostatic state. These findings support future clinical trials of PNA as an antimetastatic SOD3-mimetic drug to increase overall survival in TNBC patients.

## 1. Introduction

**Figure 1:**
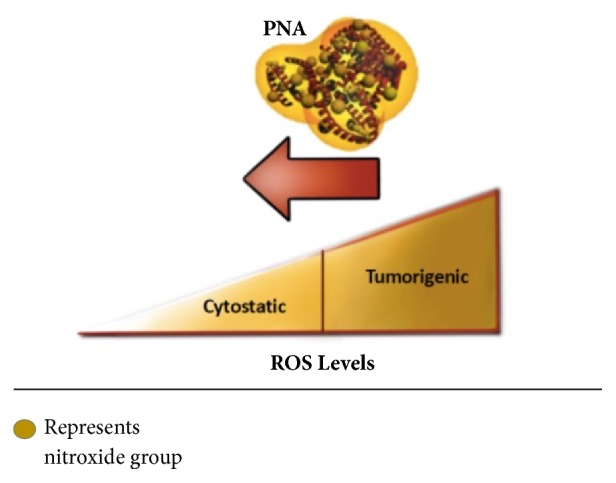
PNA is a human serum albumin that has been modified with multiple caged nitroxide groups. The nitroxide groups exhibit antioxidant activity and have the ability to catalytically remove superoxide in the vasculature and intracellular spaces. Tumors display elevated levels of reactive oxygen species that promote growth and metastasis. PNA is expected to reduce tumor ROS, leading to inhibitory effects on tumor growth, metabolism, and metastasis.

The development of antimetastatic drugs is urgently needed to reduce cancer-related deaths caused by metastasis [1]. In humans, metastasis accounts for up to 90% of all cancer-related deaths [2]. For breast cancer, triple negative breast cancer (TNBC) has the greatest chance of developing distant metastases, despite existing systemic therapy with radiation, chemotherapy, and immunotherapy [3]. As there are no currently available targeted therapies for this aggressive disease, we hypothesized that a new treatment which targets the overproduction of extracellular superoxide and its related reactive oxygen species (ROS) may have a major impact on survival of these patients [4, 5]. Due to the inadequacy of a single therapy to effectively eliminate tumors and prevent aggressive regrowth, alternative therapies that target the broader tumor microenvironment (TME) are also warranted. In this study, we examine the efficacy of PNA, an extracellular superoxide dismutase (EcSOD or SOD3) mimetic that was originally developed for ischemic stroke [6, 7]. In stroke, PNA is expected to increase the Golden Hour for treatment via its remarkable ability to treat both ischemic and hemorrhagic stroke safely without neuroimaging before thrombolytic therapy. PNA works by enhancing blood flow without oxidative stress and serving as a neuroprotectant [8]. PNA is human serum albumin (HSA) that has been modified with multiple caged nitric oxide groups (Figure 1). With albumin as the carrier, PNA is distributed in the intravascular and lymph compartments to intercept metastasizing cancer cells. PNA targets the vasculature of the TME leading to the restoration of blood flow to the hypoxic tumor core, presumably through the removal of superoxide in the intravascular space. This TME activity is facilitated by the nitroxide groups that exhibit superoxide mimetic activities [6, 7, 9]. Thus, PNA has been demonstrated to catalytically dismutate superoxide in the vasculature and increase the bioavailability of endogenous nitric oxide (NO), leading to improved blood flow. Improved blood flow in tumors has been shown to reverse tumor hypoxia [10], leading to inhibitory effects on tumor growth, metabolism, and metastasis.

The design of PNA, consisting of a novel combination of a HSA and nitroxide as a metal-free superoxide dismutase (SOD3) mimetic, has advantages over small molecular weight and membrane permeable nitroxide that mimics SOD1, SOD2, and SOD3, in cancer therapy. Albumin has a number of characteristics that make it an attractive drug vehicle or* primer*, including the ability of the protein to carry hydrophobic drugs, such as paclitaxel, through the blood and deliver them directly to cancer cells [11]. With HSA as the carrier of the nitroxides, PNA is targeted to the vasculature to increase blood flow and is in equilibrium with the lymph system [7, 9]. The SOD3 mimetic activity of PNA can break down ROS, such as superoxide, and alter the ROS balance within cancer cells and the tumor microenvironment. ROS have been shown to be involved in several cellular processes including proliferation, growth, migration, and apoptosis [12]. In cancer cells, ROS plays a pivotal role in many signaling pathways involved in carcinogenesis and metastasis [13]. For example, increased extracellular superoxide radical has been associated with increased prostate cancer cell invasion [14]. Therefore, we hypothesize that the SOD mimetic activity of the nitroxide groups on PNA will reduce the metastatic phenotype of the tumors, as well as decrease the formation of new cancer cells.

The cores of tumors are often chronically hypoxic, which is closely associated with a negative prognosis and a metastatic phenotype which is more resistant to cancer therapy [15, 16]. By targeting the hypoxic core of primary and metastatic sites, PNA may act to enhance blood flow and improve the therapeutic effects of standard chemotherapy, radiotherapy, and/or immunotherapy.

Recently, it was discovered that deficiency in EcSOD or SOD3 is involved in metastasis of multiple cancer types including TNBC and pancreatic cancer [4]. It is also established that, in the tumorigenic state, the levels of superoxide radicals in the TME are elevated [17–20]. Therefore, a SOD3 mimetic, like PNA, may be suitable as a targeted therapy to replace the deficient SOD3 in cancer cells. The data reported here provides support for this hypothesis in a highly metastatic mouse 4T1 TNBC model.

In this study we demonstrate that PNA inhibits metastasis leading to improved overall survival in a highly metastatic murine model of TNBC. The mechanisms involved in this process are as follows: (1) removal of superoxide and its related ROS of cancer cells; (2) improvement in blood flow to the tumor hypoxic core; (3) reversal of metastasis induced by the superoxide producing chemotherapeutic drug, e.g., doxorubicin; (4) extension of survival when used conjunctively with three standard conjunctive chemotherapeutic drugs with concomitant inhibition of metastasis to the lung.

## 2. Materials and Methods

### 2.1. Cell Culture

The mouse mammary carcinoma line 4T1 was purchased from ATCC and used within 5 passages. Cells were cultured in DMEM with 10% fetal bovine serum (FBS), penicillin/streptomycin solution, and amphotericin and incubated at 37°C in 5% CO2. For the SYTOX® Green (ThermoScientific) cell proliferation assay, 4T1 cells were plated in 96-well plates and allowed to attach overnight before being treated with HSA or PNA at indicated concentrations in triplicate. After 72 hr of treatment, cells were stained with 20 *μ*M SYTOX® Green for 15 min. and dead cells' fluorescence was measured in a Spectromax M5 plate reader (Molecular Devices) at excitation/emission 485/530 with a 515 emission cutoff. Then the cells were permeabilized by addition of 6 % Triton-X for 30 min, and total cell fluorescence was measured at excitation/emission 485/530 with a 515-emission cutoff. Data is representative of three independent experiments.

For the colony assays, 4T1 cells were plated at 250 cells per well in a Corning® 6-well plate. After 24 hours, the cells were treated in triplicate with the indicated concentration of PNA or HSA. After 5 days of treatment, cells were washed with PBS and fixed with 70% ethanol for 5 minutes. Colonies were washed again with PBS and then stained with Coomassie Blue [40% methanol, 12% glacial acetic acid, and 0.24% Coomassie Blue]. This was followed by another wash, and then the plates were imaged using an AlphaImager System. Colony number and size were analyzed using AlphaImager System image analysis software (AlphaInnotech, Santa Clara, CA).

### 2.2. Flow Cytometry

The production of ROS was detected by using the dye 2',7'-dichlorofluorescein diacetate (DCF-DA), a cell-permeant indicator for reactive oxygen species. DCF is used as a qualitative marker for cellular oxidant stress rather than a marker for specific ROS [21]. 4T1 cells were treated with PNA or HSA at concentrations ranging from 30 to 120 *μ*M for 16-18 hours and then stained with DCF-DA (10 *μ*M). DCF fluorescence was measured using BD C sampler software on the Accuri C6 Flow Cytometer (BD Biosciences).

### 2.3. Polynitroxyl Albumin

Polynitroxylated albumin (PNA, aka Vascular Albumin with Caged Nitric Oxide or VACNO) is a drug product prepared as a sterile, nonpyrogenic preparation in saline solution. Each 100 mL PNA contains 20 g of injectable-grade human serum albumin (HSA) and 2.5 g of 4-(2-acetamido)-2,2,6,6-tetramethyl-1-piperidinyloxy (AcTPO) covalently attached to the albumin molecules with added stabilizers, including 0.08 moles of sodium caprylate and 0.08 moles of sodium acetyltryptophanate per gram of albumin.

### 2.4. Mouse Studies

Balb/c mice (Jackson Laboratory) between 4 and 6 weeks of age were maintained in a barrier facility on high efficiency particulate air (HEPA)-filtered racks. The animals were fed with autoclaved laboratory rodent diet (Envigo diet 2918). All animal studies were approved by the Sanford Institutional Animal Care and Use Committee and conducted in accordance with the principles and procedures outlined in the NIH Guide for the Care and Use of Animals.

4T1 cells (5 X 10^4^) were injected subcutaneously into the hind limb of female Balb/c mice. In the first* in vivo* experiment examining how PNA affects metastasis, 60 mice were randomly divided into the following groups: PNA (12.5 ml/kg), control HSA (10%), doxorubicin (4 mg/kg, Sigma Aldrich), and doxorubicin (4 mg/kg) plus PNA (12.5 ml/kg). When tumors became palpable (4 days), intraperitoneal (i.p.) injections with PNA, control HSA, doxorubicin, or PNA plus doxorubicin were performed. PNA and HSA treatments were performed three times a week.

In the second* in vivo* 4T1 experiment, 50,000 4T1 cells were subcutaneously injected into Balb/c mice, and 45 mice were divided into the following groups: (1) control chemotherapy group (15 mice), (2) chemotherapy plus PNA delivered intraperitoneally (i.p.) (15 mice), and (3) chemotherapy plus PNA delivered intravenously (i.v.) (15 mice). When tumors became palpable (4 days), the groups received the following treatments: in weeks 1-2, control group received HSA combined with paclitaxel (60 mg/kg), paclitaxel (60 mg/kg) plus PNA delivered i.p., and paclitaxel plus PNA delivered i.v. and in weeks 3-4, the control group received HSA plus doxorubicin (4mg/kg) plus cyclophosphamide (100mg/kg). The experimental groups received doxorubicin, cyclophosphamide (100 mg/kg) and PNA delivered i.p. and doxorubicin, cyclophosphamide, and PNA delivered i.v. The paclitaxel, doxorubicin, and cyclophosphamide were administered once a week, and PNA and has were delivered 3 times a week.

Lung metastasis was quantified in 4T1 mice through injection of India ink into whole lungs via the trachea following sacrifice of the animals as described [22, 23]. The number of visible metastases was manually counted using a dissecting microscope (Nikon SMZ1000).

Quantification of necrotic areas of primary tumor sections was performed using stereology methodology. At the time of sacrifice, primary tumors were harvested, fixed in 10% buffered formalin, and paraffin embedded (FFPE). Tumors were sectioned to 5 *μ*m. Hematoxylin and Eosin (H & E) staining was performed, and analysis of live and dead tissue on tumor sections was performed using Stereo Investigator software (version 10, Micro Bright Field; Williston, VT) by outlining areas of karyolysis indicated by reduced number of nuclei and increased eosin staining. Briefly, the tumor area was outlined, and a grid of counting frames was applied to systematically quantify across the entire tumor section. The area of live tumor tissue versus nonviable was calculated using the area fraction fractionator probe. Counting frames of 200 *μ*m by 200 *μ*m were placed over the tumor section with an average of 50 counting frames per tumor. Tumor tissue was defined as viable or necrotic and the corresponding area of each was calculated using the software package.

### 2.5. Ultrasound Measurement of Tumor Perfusion

The day prior to ultrasound imaging, a depilatory cream (Nair) was applied to skin on the tumor and surrounding regions to prevent interference with the ultrasound transducer. On the day of imaging, animals were anesthetized with 1.5% isoflurane in oxygen, placed on a heated stage, and restrained using surgical tape. Anesthesia was maintained during imaging using 1.5% isoflurane in oxygen administered via nose cone. Warmed ultrasound gel (Parker Laboratories) was applied to the depilated skin and ultrasound images were captured (Vevo2100; Visual Sonics).

To measure tumor vascularity, Color Doppler imaging was performed on 4T1 tumors 14 days after injection. Tumors had been treated starting 4 days after initiation with PNA or control HSA 3 times per week as described above. Color Doppler imaging was performed across the entire tumor using serial scans. After image-acquisition, the tumor was outlined on the serial scans and the volume was calculated and percent of vascularity within the outlined tumor was calculated by manufacturer's software (Visual Sonics).

To calculate tumor perfusion time following administration of PNA, a microbubble contrast agent (Vevo MicroMarker; Visual Sonics) was used. Tumors had been treated starting 4 days after initiation with PNA or control HSA 3 times per week as described above. Tumor perfusion studies were performed 16 days after tumor initiation. The following procedures were performed in addition to those mentioned above: a 27-gauge butterfly needle connected to a catheter was inserted via tail vein for intravenous injection of the microbubbles contrast agent. The microbubble contrast agent was prepared according to manufacturer instructions and a bolus injection of 50 *μ*L was delivered via tail vein catheter flushed with 20-30 *μ*L of saline. Perfusion of the tumor microvasculature was calculated using manufacturer software (VevoCQ; Visual Sonics). A baseline perfusion prior to administration of PNA or control HSA was obtained. Following baseline acquisition, PNA (12.5 mL/kg) or control HSA (10%) were delivered via tail vein injection. Thirty minutes after drug injection, a second dose of microbubbles was administered and tumor perfusion was calculated as described above. Tumor perfusion following drug delivery was calculated by taking the 30-minute perfusion time minus the baseline perfusion time.

***Statistics.*** Data is expressed as means ±SEM. P values were determined by paired student's t-tests.

## 3. Results

### 3.1. PNA Reduces Reactive Oxygen Species

**Figure 2:**
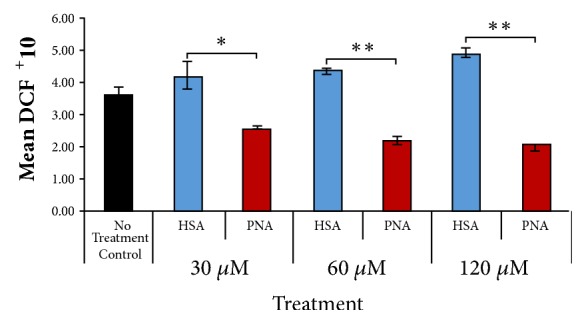
*Treatment with PNA reduces intracellular reactive oxygen species.* 4T1 cells were treated with PNA or corresponding concentration of control HSA for 16-18 hours and then stained with 2',7'-dichlorofluorescein diacetate and measured via flow cytometry. In the presence of reactive oxygen species (ROS), the dye is converted to the fluorescent molecule 2',7'-dichlorofluorescein (DCF) (n=3 for each group; data is representative of three independent experiments; *∗* = p < 0.05, *∗∗* = p < 0.01 versus HSA).

The key property of PNA is its antioxidant activity, which is mediated by catalytic dismutation of superoxide and related ROS. We hypothesized that PNA treatment of tumor cells would reduce cellular ROS levels, leading to changes in growth and survival. To test this, cultured 4T1 cells were treated with PNA (30-120 *μ*M) for 24 hours. Control cultures received the same concentrations of HSA, the protein component of PNA. Cellular ROS levels were estimated by staining cells with DCF-DA. At each concentration, 4T1 cells treated with PNA had significantly reduced ROS as compared to the corresponding control HSA treatment (Figure 2). Thus, PNA potentially shifts 4T1 cancer cells from a tumorigenic to a cytostatic state (see Figure 1).

### 3.2. PNA Treatment of Tumor-Bearing Animals Shows Reduced Tumor Perfusion Time and Increased Relative Blood Volume Compared to Control in a Breast Cancer Mouse Model

**Figure 3:**
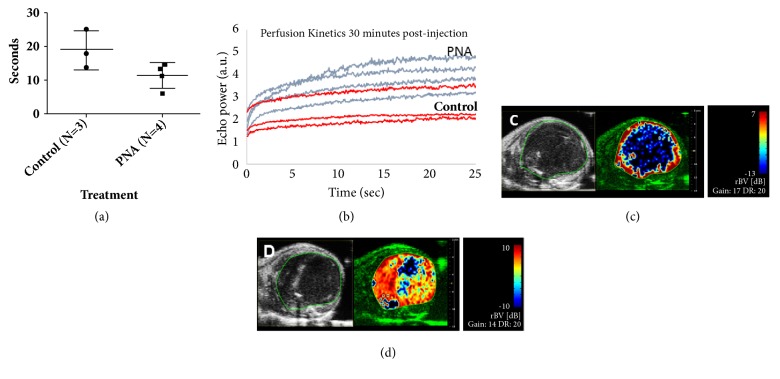
*PNA treated tumors have reduced tumor perfusion time and increased relative blood volume in the metastatic TNBC 4T1 model.* Ultrasound imaging was used to visualize tumor microvasculature. The contrast agent was administered intravenously, images were acquired, and perfusion time was calculated with manufacturer software. PNA or HSA control was administered intravenously. And after 30 minutes, a second dose of contrast agent was administered, and perfusion time was calculated. (a) PNA treated tumors have reduced tumor perfusion time compared to HSA control. Tumor perfusion time was calculated by taking the 30-minute perfusion time minus the baseline perfusion time. (b) The kinetics of perfusion as measured by echo power versus time indicate PNA treated tumors have higher echo power at peak perfusion time compared to HSA control, indicating increased blood flow within the tumor. (c) Tumors were outlined in ultrasound B-mode (left panel). Parametric imaging of relative blood volume is shown 30 minutes after drug injection at the time of peak perfusion (right panel). HSA treated tumors show little to no signal intensity in the core of the tumor. (d) PNA treated tumors show greater relative blood volume within the tumor core.

Our previous studies show that PNA promotes blood flow in animal models of cerebral ischemia, leading to significant reductions in infarct size [6, 7]. Similar findings have been found in cardiac ischemia reperfusion models [24]. This activity is mediated by dismutation of superoxide in blood vessels, preventing the formation of peroxynitrite and allowing the restoration of endogenous nitric oxide in the vasculature. We have recently reported that the restoration of blood flow by PNA in acute hypoxia arising in ischemia stroke also restores blood flow in chronic hypoxia in solid tumors [22]. PNA was shown to promote blood flow and drug delivery to hypoxic flank tumors in a xenograft tumor model using electron paramagnetic resonance (EPR) measurements [22]. To confirm that PNA is also effective in opening up the 4T1 TNBC flank hypoxic tumor core, we tested whether PNA increased tumor blood flow using ultrasound bursting and imaging techniques.  Figure 3(a) shows ultrasound measurements demonstrating that PNA increased the perfusion rate of the hypoxic core of 4T1 flank tumors compared to the tail vein infusion of HSA.  Figure 3(b) shows the reperfusion kinetics of the microbubble burst by the ultrasound. Figures 3(c) and 3(d) illustrate the difference of the perfusion volume of the flank tumor detected by the microbubble technique from tail vein infusion of HSA versus PNA, respectively. These ultrasound measurements together with the EPR measurements independently validated that the SOD3-mimetic activity PNA improves blood flow within the hypoxic core of two types of solid tumors.

### 3.3. PNA Is Cytotoxic and Inhibits Cancer Cell Proliferation in TNBC

**Figure 4:**
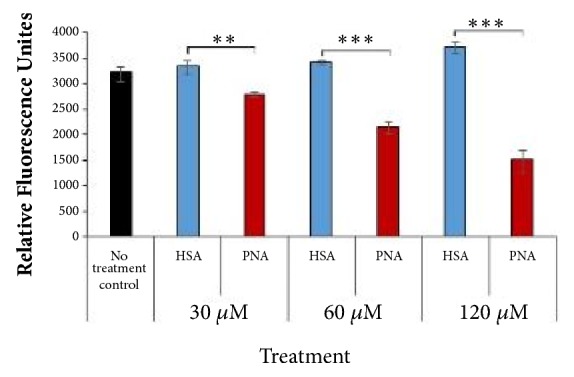
*PNA treatment significantly reduces live cell number.* 4T1 cells were plated and treated the following day with PNA (red bars) or HAS (blue bars), and control (black bar) was media only. After 72 hr, cells were stained with SYTOX® Green and fluorescence was measured. Percent of dead cells and percent of live cells were calculated using fluorescence values. Live cells' fluorescence is shown. Results indicate PNA, compared to HSA, reduces live cell numbers at concentrations ranging from 30 to 120 *μ*M PNA (n=3 for each group; *∗∗*=p<0.01, *∗∗∗*= p<0.001).

It is well known that elevated levels of ROS are protumorigenic through activation of signaling pathways that promote cell proliferation, cell survival, and oncogenic transcriptional programs [12–14]. Since PNA reduces cancer cell ROS in 4T1 cells (Figure 2), we predicted that PNA would also reduce proliferation of 4T1 cells. In order to examine this hypothesis, PNA was tested* in vitro* in 4T1 mouse TNBC cell cultures. In this dose response study, cells were exposed for 72 hr to 30 *μ*M, 60 *μ*M, and 120 *μ*M of PNA or corresponding concentrations of control HSA. Live and dead cell numbers were determined using a SYTOX® Green assay, and live cell number was then extrapolated from this data (Figure 4). PNA compared to HSA significantly reduced the number of live cells in a dose dependent manner (n=3 for each group, *∗∗*=p<0.001; *∗∗∗*=p<0.0001).

**Figure 5:**
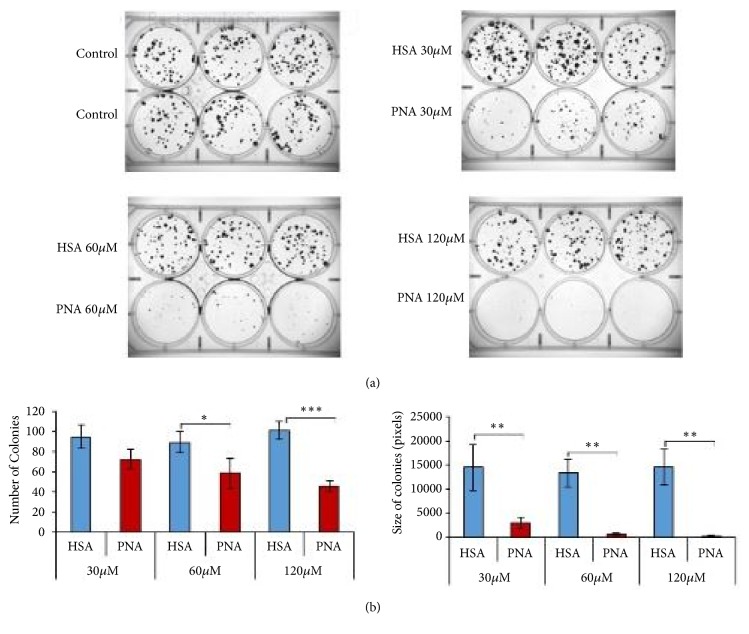
*PNA treatment significantly reduces TNBC cancer cell proliferation*. Colony forming assays were performed in PNA treated 4T1 cell cultures. Cells were plated at low density and then treated with the indicated concentration of PNA (red bars) or HSA (blue bars) as a control. PNA significantly reduces number of colonies compared to HSA treatments (a) and size of colonies (b). n=3 per group; data is representative of three independent experiments (*∗* = p < 0.05, *∗∗* = p < 0.01, and *∗∗∗* = p < 0.001 versus HSA).

The effect of PNA on cell proliferation and survival was further examined in colony formation assays. 4T1 cells were studied for their ability to form colonies within 5 days in the presence of varying doses of PNA and HSA as in Figure 4. In Figure 5, colonies were stained and imaged, and the number and size of the colonies were quantified. There was a significant dose dependent decrease in the number (Figures 5(a) and 5(b), left panel) and size of colonies (Figures 5(a) and 5(b), right panel) in response to PNA compared to control HSA at concentrations ranging from 30 to 120 *μ*M (p <0.001). Results from Figures 4 and 5 suggest that PNA inhibited proliferation and colony formation of 4T1 cells by reducing the superoxide and ROS levels.

### 3.4. Small Molecule, Membrane Permeable Nitroxide Compound Affects 4T1 Growth and Survival Differently Compared to PNA

**Figure 6:**
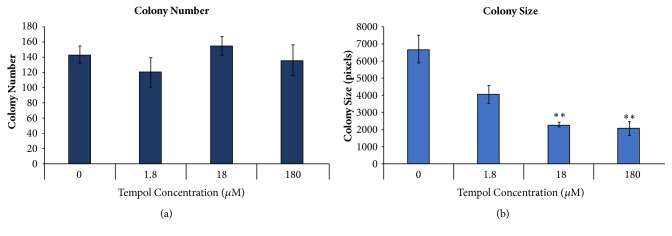
*TEMPOL produces significant decreases in colony size but not colony number.* 4T1 cells were plated at low cell number and treated the following day with TEMPOL at varying concentrations, and the control was DMEM alone. After 5 days, cells were fixed, stained, and imaged using Alpha Imager software. (a) Colony number was counted and (b) total colony size was measured using Alpha Imager software. Data shown suggests that TEMPOL alone inhibits cellular proliferation at concentrations ranging from 18 to 180 *μ*M, according to a two-tailed t test, with p <0.001, but is not significantly cytotoxic at the concentrations shown.

Macromolecular PNA is primarily extracellular and, therefore, is an EcSOD/SOD3 mimetic. TEMPOL (4-hydroxy-2,2,6,6-tetramethylpiperidine-N-oxyl), on the other hand, is a small molecule nitroxide that is membrane permeable, reaching all cellular compartments. A dose response study showed that TEMPOL significantly inhibits the 4T1 colony size, at concentrations ranging from 18 to 180 *μ*M, suggesting cytostatic effects on the cancer cells (Figure 6(a)). However, the TEMPOL did not significantly reduce colony number (Figure 6(b)), suggesting a lack of cytotoxic effects on the cancer cells. These results indicate that PNA and small molecule, membrane permeable nitroxides have differences in their specificities and therapeutic indices.

**Figure 7:**
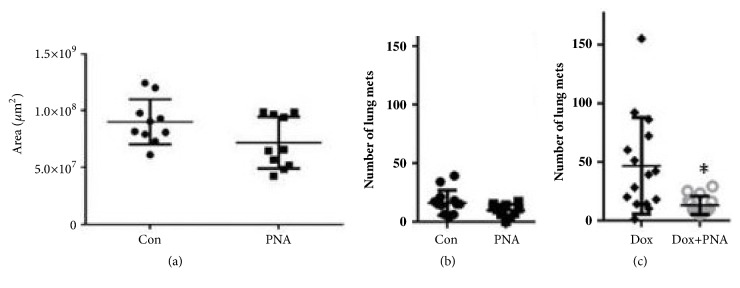
*Live tumor area and number of metastatic lung nodules decreases with PNA treatment while survival increases with PNA treatment.* (a) Subcutaneous 4T1 tumors are treated with HSA (control) or PNA. Tumors sections were stained by H&E and live tissue area was determined by stereology. (b) Tumor bearing mice were treated with or without PNA and after 3 weeks lungs were harvested for counting lung metastatic nodules. (c) 4T1 tumor bearing mice were treated with doxorubicin in the presence or absence of PNA. PNA combined with doxorubicin compared to doxorubicin alone significantly reduces lung metastasis, according to a paired two-tailed t test, p <0.05 (*∗*).

***Effects of PNA on 4T1 tumors in vivo.***In vivo, 4T1 cells establish rapidly growing tumors that metastasize to lung, liver, bone, and brain [25]. Thus, they are a useful model of human TNBC. Subcutaneous 4T1 tumors were developed in Balb/c mice and treated with PNA or HSA as a control. Tumors from PNA treated mice were not significantly different in size from those of HSA treated animals. However, histological examination of tumors from both groups showed that PNA treated tumors had less live tissue and more necrotic (dead) tissue than the HSA treated controls (Figure 7(a)). The difference between control and PNA treated groups was approaching significance (p= 0.0705) but suggested that PNA is having a direct effect on primary tumors as well as on metastasis.

### 3.5. PNA Reduces Lung Metastasis from 4T1 Primary Tumors

**Figure 8:**
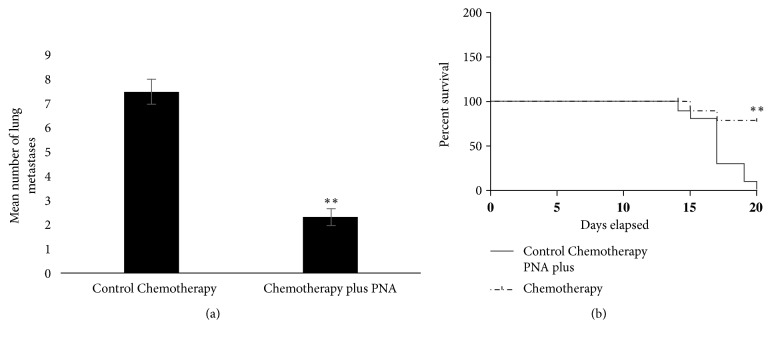
*PNA treatment combined with chemotherapy reduces lung metastasis and enhances survival of mice carrying 4T1 tumors compared to chemotherapy treatment alone.* (a) Tumor bearing mice were treated with a chemotherapy regimen, with and without PNA, and after 3 weeks lungs were harvested for counting metastatic lung nodules. PNA in the presence of chemotherapy significantly reduced lung metastases compared to chemotherapy alone, according to a paired two-tailed t test, p >0.01. (b) PNA combined with chemotherapy significantly increased overall survival of mice compared to chemotherapy alone, according to the Log-Rank (Mantel-Cox) test, with p value =.0013 (*∗∗*).

Subcutaneous or orthotopic 4T1 tumors rapidly metastasize to distant tissues in a pattern similar to human TNBC, including metastasizing to the lung [25, 26]. We found that PNA reduced lung metastasis as compared to control HSA treatment but not to a level of significance (Figure 7(b), p=0.056). However, we found that there is a dramatic increase in metastasis to the lung induced by a superoxide producing chemotherapy drug doxorubicin (Figure 7(c)). Remarkably, in the presence of PNA and doxorubicin the lung metastasis was nearly abolished (Figure 7(c), paired two-tailed t test, p=0.038). This provides strong rationale for combining PNA with other drugs used in adjuvant or neoadjuvant therapy. In this regard, mice bearing 4T1 tumors were subjected to a chemotherapy regimen including paclitaxel, doxorubicin, and cyclophosphamide, with or without PNA (Figure 8(a)). This regimen is similar to that in the I-SPY 2 clinical trials for breast cancer [27]. In this study, PNA again significantly decreased the number of lung metastases (paired t test with p<0.01). Furthermore, PNA enhanced overall survival of mice against chemotherapy-mediated toxicity in the 4T1 mouse model (Figure 8(b)). Statistical analysis using the Log-Rank (Mantel-Cox) test indicates a statistical difference between PNA and chemotherapy treated mice compared to HSA and chemotherapy treated mice, with p<0.0013. Together, these data demonstrate that PNA reduces metastasis in this triple negative breast cancer model and that this correlates with longer survival times. Thus, this indicates PNA conjunctive therapy with standard chemotherapeutic agents improves survival of 4T1 tumor bearing mice corresponding to a reduction in toxicity of the chemotherapeutic agent and a reduction in lung metastasis.

## 4. Discussion

Our findings demonstrate that the SOD3-mimetic activity of an extracellular albumin-based macromolecular nitroxide, PNA, converts cancer cells from a tumorigenic state to a cytostatic state by reducing superoxide and ROS levels (see Figure 1). Earlier we reported that PNA works as an SOD3 mimetic to alter the hypoxic state of a solid tumor through blood flow enhancement [28]. In this report we established that the same therapeutic mechanism for PNA also works in the 4T1 TNBC model. We were able to confirm that this action of PNA occurs within 30 minutes of i.v. infusion with two independent biophysical methods (EPR and ultrasound).

Our data suggest that PNA is cytotoxic to murine 4T1 TNBC cells as well as inhibiting their proliferation. In colony forming assays, PNA reduced the number of colonies, suggesting an effect of PNA on the clonogenic survival capacity of cells. PNA also reduced the size of the colonies, indicating reduced ability of the cancer cells to proliferate. More importantly, we have demonstrated that PNA inhibits metastasis, particularly that induced by chemotherapeutic drugs such as doxorubicin, leading to enhanced survival (Figures 7 and 8). We discuss below the significance of the current results for future preclinical and clinical studies that can guide the development of PNA as a* primer* used in conjunction with standard cancer therapies. Thus, our preclinical studies may be relevant to neoadjuvant treatment and to clinical trials such as the I-SPY2 trials [27].

Two key aspects of PNA facilitate its biological activities. First, it catalyzes the dismutation of superoxide by virtue of the multiple covalently attached nitroxide groups. Second, it is based on a macromolecule carrier, HSA, which is capable of targeting extracellular compartments. These properties of PNA are expected to create an essentially superoxide-free milieu within the vasculature and within the tumor microenvironment. In the vasculature, elimination of superoxide prevents the formation of peroxynitrite through its reaction with NO. This allows the restoration of endogenous NO to promote blood flow to the metastasized solid tumor. This is supported by our published studies that demonstrate PNA mediation of increased blood flow and prevention of ischemic reperfusion injury in models of stroke, myocardial infarction, and sickle cell disease [6, 29].

Enhanced blood flow, as shown here and in our previous study [28], is likely to have a number of effects on solid tumors. It is expected to increase oxygen delivery to tumor cells, which would likely reverse hypoxia leading to inactivation of hypoxia inducible factor 1*α*. This in turn would alter tumor metabolism and growth, as well as several aspects of metastasis including angiogenesis, migration, invasion, and intravasation [30].

PNA reduces cellular ROS in 4T1 breast cancer cell cultures and we have shown that this corresponds to reduced cell proliferation and survival. Because PNA is based on HSA, it is expected to target extracellular spaces within the tumor microenvironment. We did not observe reduced 4T1 tumor sizes in mice treated with PNA. However, the tumors did have reduced amount of live tissue (Figure 7(a)) and increased areas of noncellular or necrotic tissue (not shown), indicating that the drug was directly acting on the tumor. PNA in the extravascular spaces within the tumor microenvironment is expected to reduce tumor superoxide and ROS levels and this may directly lead to interference with metastasis. For example, ROS are able to directly activate matrix metalloproteinases (MMPs) [31]. MMPs are key effectors of tumor invasion and metastasis through degradation of extracellular matrix components. Therefore, PNA may directly interfere with metastasis by blocking MMP activation by eliminating superoxide within the tumor microenvironment. This proposed mechanism may also relate to the effects of doxorubicin and other chemotherapy reagents on metastasis. For example, we found that doxorubicin increases the number of lung metastases compared to control mice, and this was reversed by PNA. This is consistent with other studies showing doxorubicin-induced increases in metastasis in mouse models and human patients [32, 33]. Many chemotherapy agents, including doxorubicin, increase tumor superoxide and ROS, which is associated with the process of metastasis. We show that PNA eliminates the increase in metastasis associated with chemotherapy. This is most likely related to the ability of PNA to eliminate superoxide and ROS in the tumor microenvironment.

Since our experimental design has been to inject PNA into the intraperitoneal cavity, the subsequent biodistribution of PNA warrants consideration. The overall biodistribution of PNA in our studies is expected to be similar to endogenous serum albumin since PNA consists of macromolecular human serum albumin conjugated with multiple nitroxide groups to provide the SOD mimetic activity. Endogenous albumin is mainly synthesized in the liver, enters the circulation, and occurs predominantly in the extravascular space in tissues such as skin, gut, muscle, and other fluids such as cerebrospinal fluid. A substantial amount of albumin is also found within the intravascular space, but only a very small amount is thought to exist intracellularly [34–36]. In our studies, after injection into the intraperitoneal space, PNA is expected to enter the circulation via the lymphatic system and be distributed similarly to endogenous albumin. Imaging studies confirm that fluorescent albumin injected into the intraperitoneal space distributes maximally to the spleen, livers, lungs, and other organs within 3 hrs of injection; levels subsequently decline [37]. Endogenous albumin cycles through the lymphatic system approximately 28 times during its lifetime (Peters, 1996; Evans, 2002) and injected PNA is expected to behave similarly. More importantly, PNA is not expected to accumulate significantly intracellularly in vivo, similar to endogenous albumin. Thus, the activity of PNA is most similar to ecSOD/SOD3, which is also localized extracellularly.

The intraperitoneal administration of PNA may have two effects. First, PNA would remove extracellular superoxide in all bodily compartments working as EcSOD/SOD3 with minimal effect on the intracellular superoxide levels controlled by SOD1 and SOD2. In contrast a low molecular weight nitroxide chemical like TEMPOL would dismutate both intracellular and extracellular superoxide because it readily crosses membranes and accumulates within cells (Figure 6). Second, it has been known for over four decades that the lymphatics are the first site of metastasis for most solid cancers and there has been a concerted effort in trying to target cancer therapeutics to the lymphatic system (e.g., see [38]). Accumulation of PNA in the lymph nodes and surrounding lymphatics is a possibility and may affect immune cells and metastasis. The possible immunodulatory effects on metastasis by PNA will be evaluated in future studies.

As a macromolecule with multiple nitroxide groups, PNA acts as an SOD3 mimetic. SOD3 (aka EcSOD) is secreted from cells and is found either free in the extracellular space or adhered to the surface of cells. SOD3 expression is frequently diminished in breast and other types of cancer, which contributes to metastasis [4]. Furthermore, low expression of SOD3 in breast cancer is associated with reduced relapse free survival in all types of breast cancer [4]. Overexpression of SOD3 in a human breast cancer xenograft or in the 4T1 syngeneic model led to reduced lung metastasis and longer survival [39, 40].

Taken together, PNA appears to be capable of acting as an SOD3 mimetic to alter the oxidative state within solid tumors, inhibiting their ability to metastasize (see Figure 1). We speculate that elimination of superoxide within the tumor microenvironment directly reverses ROS stimulated metastasis by preventing activation of MMPs, reducing invasion, and inhibiting cancer cell proliferation. PNA enhancement of blood flow is expected to reverse solid tumor hypoxia leading to changes in tumor metabolism and hypoxia-dependent metastatic events. Another potential outcome of increased blood flow in tumors is to act as a* primer* to improve drug delivery to tumors to enhance the therapeutic index of chemotherapy and immunotherapy. An additional advantage of PNA treatment of cancer may be attributable to its ability to protect normal tissues from oxidative stress and inflammatory injuries leading to better quality of life. We have previously demonstrated the ability of PNA to prevent ischemic reperfusion injury, to reduce inflammatory injuries, and to protect normal tissue from ionizing radiation [7, 29]. In the present TNBC studies, we have observed that PNA prevents weight loss promoted by the chemotherapy drug doxorubicin (not shown), suggesting that PNA has protective effects in normal tissues. Ongoing studies are exploring this aspect of PNA as well as the detailed mechanism by which it inhibits metastasis, as well as its effectiveness in other types of solid tumors. As a novel SOD mimetic drug, PNA would be ideally suited for evaluation in trials like the I-SPY 2 platform [27] and FDA breakthrough therapy designation (BTD) to bring PNA from bench to bedside in 3-5 years.

## 5. Conclusions

PNA reduces ROS in TNBC, is cytotoxic, and is cytostatic to mouse TNBC breast cancer cells in a dose dependent manner. PNA combined with doxorubicin, compared to doxorubicin alone, significantly reduces lung metastasis in a mouse model of breast cancer. Furthermore, PNA reduces metastasis and enhances survival of mice against chemotherapy-mediated toxicity in a 4T1 mouse breast cancer model. These* in vitro* and* in vivo* experimental findings suggest PNA is a promising cancer therapeutic. The antimetastatic mechanism of the SOD3-mimetic PNA on a murine TNBC 4T1 mouse model has been established. If the same antimetastatic mechanisms can be established with a human TNBC mouse model, these preclinical efficacy data will guide a Phase 1b clinical protocol design in recurrent TNBC patients. In addition, PNA could be tested in trials like the I-SPY2 fast track clinical trial protocol to select and graduate new drug candidates through FDA breakthrough therapy designation (BTD).

## Data Availability

The data used to support the findings of this study are included within the article.
